# Effect of a 3-Week Multidisciplinary Body Weight Reduction Program on the Epigenetic Age Acceleration in Obese Adults

**DOI:** 10.3390/jcm11164677

**Published:** 2022-08-10

**Authors:** Antonello E. Rigamonti, Valentina Bollati, Chiara Favero, Benedetta Albetti, Diana Caroli, Laura Abbruzzese, Silvano G. Cella, Alessandro Sartorio

**Affiliations:** 1 Department of Clinical Sciences and Community Health, University of Milan, 20129 Milan, Italy; 2 EPIGET Lab, Department of Clinical Sciences and Community Health, University of Milan, 20122 Milan, Italy; 3 Occupational Health Unit, Fondazione IRCCS Ca’ Granda Ospedale Maggiore Policlinico, 20122 Milan, Italy; 4 Experimental Laboratory for Auxo-Endocrinological Research, Istituto Auxologico Italiano, IRCCS, 28824 Piancavallo-Verbania, Italy; 5 Division of Auxology and Metabolic Diseases, Istituto Auxologico Italiano, IRCCS, 28824 Piancavallo-Verbania, Italy

**Keywords:** obesity, biological age, DNA methylation, epigenetic age acceleration, body weight reduction program, age-related diseases, obesity-related diseases

## Abstract

Obesity and aging share common molecular and cellular mechanisms underlying the pathophysiology of cardiovascular diseases (CVD), which occur frequently in both conditions. DNA methylation (DNAm) age, a biomarker of the epigenetic clock, has been proposed as a more accurate predictor of biological aging than chronological age. A positive difference between an individual’s chronological age and DNAm age is referred to as epigenetic age acceleration. The objective of the present study was to evaluate the effects of a 3-week in-hospital body weight reduction program (BWRP) on the epigenetic age acceleration, as well as on other cardiometabolic outcomes, in a cohort of 72 obese adults (F/M: 43/29; (chronological) age: 51.5 ± 14.5 yrs; BMI: 46.5 ± 6.3 kg/m^2^). At the end of the BWRP, when considering the entire population, BMI decreased, and changes in body composition were observed. The BWRP also produced beneficial metabolic effects as demonstrated by decreases in glucose, insulin, HOMA-IR, total cholesterol, and LDL cholesterol. A post-BWRP improvement in cardiovascular function was also evident (i.e., decreases in systolic and diastolic blood pressures and heart rate). The BWRP reduced some markers of systemic inflammation, particularly C-reactive protein (CRP). Finally, vascular age (VA) and Framingham risk score (FRS) were reduced after the BWRP. When considering the entire population, DNAm age and epigenetic age acceleration did not differ after the BWRP. However, when subdividing the population into two groups based on each subject’s epigenetic age acceleration (i.e., ≤0 yrs or >0 yrs), the BWRP reduced the epigenetic age acceleration only in obese subjects with a value > 0 yrs (thus biologically older than expected). Among all the single demographic, lifestyle, biochemical, and clinical characteristics investigated, only some markers of systemic inflammation, such as CRP, were associated with the epigenetic age acceleration. Moreover, chronological age was correlated with DNAm age and VA; finally, there was a correlation between DNAm age and VA. In conclusion, a 3-week BWRP is capable of reducing the epigenetic age acceleration in obese adults, being the BWRP-induced rejuvenation evident in subjects with an epigenetic age acceleration > 0 yrs. Based on the BWRP-induced decrease in CRP levels, chronic systemic inflammation seems to play a role in mediating obesity-related epigenetic remodeling and biological aging. Thus, due to the strong association of CVD risk with the epigenetic clock and morbidity/mortality, any effort should be made to reduce the low-grade chronic inflammatory state in obesity.

## 1. Introduction

Obesity and aging have been provocatively defined as “two sides of the same coin” [1].

Obesity increases the risk of premature death by about 1.5 to 2.5-fold and shortens lifespan by up to 20 years [2,3], thus making us speculate that obesity may accelerate aging [4]. In particular, obese adults are at higher risk of a huge number of typically age-related diseases such as cardiovascular diseases (CVD), blood hypertension, dyslipidemia, type 2 diabetes mellitus (T2DM), neurodegenerative diseases, osteoarticular diseases, sarcopenia, and cancer [5].

From the view of cell biology, by using different DNA-methylation-based estimators of the epigenetic clock, an epigenetic age acceleration has been documented in different tissues from animal and human obesity, including peripheral leucocytes [6].

These findings are not surprising, because obesity and aging share a similar spectrum of pathophysiological mechanisms promoting age-related changes in DNA methylation (DNAm) until cellular senescence: low-grade systemic inflammation, redox imbalance, mitochondrial dysfunction, accumulation of cytotoxic macromolecules, and impairment of the immune system [7].

Fortunately, as shown by animal and human studies, obesity- and age-related diseases seem, at least partially, reversible when appropriate interventions are adopted early [8,9].

In this regard, caloric restriction (CR), which represents a fundamental component of any body weight reduction program (BWRP) administered to obese patients [10,11], is characterized by a reduction in calorie intake without causing malnutrition. Apart from the beneficial effects on cardiometabolic outcomes, for a long time, CR has been recognized as the only non-pharmaceutical and non-genetic intervention capable of delaying the onset of age-related diseases and extending the lifespan in a range of experimental models, including both normal weight and obese humans [12]. Many cellular and molecular mechanisms have been proposed to explain the CR-induced anti-aging (rejuvenation?) effects, such as the improvement of mitochondrial bioenergetics with decreased oxidative damage, inhibition of insulin-growth factor 1 (IGF-1), and mammalian target of rapamycin (mTOR) dependent pro-aging signaling, and anti-inflammatory actions at systemic level [13]. Epigenetic remodeling has been invoked to play a crucial role as suggested by the ability of CR to change DNAm status [14].

Exercise, which is often combined with CR in multidisciplinary BWRP, has been demonstrated to have an anti-aging effect [15]. In particular, similar to CR, exercise reduces systemic inflammation and oxidative stress, two pathophysiological mechanisms that have been reported to accelerate epigenetic age [16,17].

Given the strong evidence that epigenetic remodeling, a “reversible” molecular process, contributes to determining lifespan by predisposing to obesity- and age-related diseases [18], we have decided to verify whether a 3-week multidisciplinary BWRP, including CR and exercise, was capable, in a cohort of severely obese adults, of suppressing age-associated changes in DNAm. That is, to reduce the epigenetic age acceleration as calculated by using a mathematical algorithm based on the modern DNAm array technology, which enables the identification of the specific genomic locations of CpGs. In particular, we conceptualized an estimator of epigenetic age based on five sets of CpGs (also known as “clock CpGs”) that have been reported to accurately predict the “biological” age (in units of years) of a DNA source, such as peripheral leucocytes [19]. In physiological conditions, epigenetic age (or DNAm age) should coincide with chronological age (i.e., epigenetic age acceleration equal to zero), but, in pathological conditions like obesity, an epigenetic age acceleration (i.e., epigenetic age > chronological age) has been reported [4].

We hypothesize that BWRP might be effective in reducing the epigenetic age acceleration and that the BWRP-induced slowing of the epigenetic clock might be more evident in obese subjects characterized by more accelerated aging.

The results of our clinical study might shed some light on the intricate connections between obesity and aging and pave a new path toward developing therapeutic strategies against obesity-related diseases, including CVDs and even cancer.

## 2. Materials and Methods

### 2.1. Subjects

A set of adults were selected from the obese patient population admitted to the Division of Metabolic Diseases of the Istituto Auxologico Italiano, Piancavallo (VB), Italy, for a 3-week in-hospital multidisciplinary BWRP.

The inclusion criteria were individuals of both sexes, aged > 18 yrs, having a body mass index (BMI) > 35 kg/m^2^, with or without metabolic syndrome (see below for its definition), independently of the level of physical activity. The exclusion criteria were: (1) secondary causes of obesity (e.g., Prader–Willi syndrome, steroid-induced or medication-induced obesity); (2) individuals with systolic blood pressure (SBP) ≥ 180 mmHg and diastolic blood pressure (DBP) ≥ 110 mmHg; (3) CVDs clinically evident in the previous six months; and (4) individuals who refused to sign the consent form.

After clinical evaluation, based on the above-reported inclusion/exclusion criteria, participants were recruited and, after the completion of the 3 weeks of BWRP, were included in the statistical analysis. 

Some of these were taking medications for diabetes (metformin and/or insulin), hypertension, and dyslipidemia (see the Results for details). 

The protocol was approved by the Ethical Committee of the Istituto Auxologico Italiano (research project code: 01C022; acronym: ETABIOLOB); the protocol was explained to the patients, who gave their written informed consent.

### 2.2. Body Weight Reduction Program (BWRP)

The BWRP consisted of a 3-week in-hospital integrated energy-restricted diet (1200–1800 kcal/day) in combination with physical rehabilitation (moderate aerobic activity), psychological counseling, and nutritional education. The amount of energy to be given with diet was calculated by subtracting approximately 500 kcal from the measurement of resting energy expenditure, which was determined after an overnight fast through open-circuit, indirect computerized calorimetry (Vmax 29, Sensor Medics, Yorba Linda, CA, USA) with a rigid, transparent, ventilated canopy. The diet, in terms of macronutrients, contained 21% proteins, 53% carbohydrates, and 26% lipids; the daily estimated water content was 1000 mL, while the estimated salt content was 1560 mg Na^+^, 3600 mg K^+^, and 900 mg Ca^2+^. Extra water intake of at least 2000 mL/day was encouraged.

The physical activity program consisted of five days per week of training, including (i) 1 h dynamic aerobic standing and floor exercise with arms and legs, at moderate intensity and under the guidance of a therapist; and (ii) either 20–30 min cycle ergometer exercise at 60 W, or 3–4 km out-door walking on flat terrain, according to individual capabilities and clinical status. 

The subjects also underwent a psychological counseling program consisting of two or three sessions per week of individual and/or group psychotherapy performed by clinical psychologists. Furthermore, lectures, demonstrations, and group discussions, with or without a supervisor, took place daily.

### 2.3. Anthropometric Measurements

A scale with a stadiometer was used to determine height and weight (Wunder Sa.Bi., WU150, Trezzo sull’Adda, Italy). Waist circumference (WC) was measured with a flexible tape in a standing position, halfway between the inferior margin of the ribs and the superior border of the crista, while hip circumference (HC) was measured at the largest parts around the buttocks. Body composition was measured by bioimpedance analysis (Human-IM Scan, DS-Medigroup, Milan, Italy) after 20 min of supine resting. BMI, fat mass (FM), and fat-free mass (FFM) were determined in all subjects.

### 2.4. Metabolic Variables

Blood samples (about 10 mL) were collected at around 8:00 a.m. after an overnight fast at the beginning and the end of the BWRP (i.e., at T1 and T2). 

Total cholesterol (T-C), high-density lipoprotein cholesterol (HDL-C), low-density lipoprotein cholesterol (LDL-C), triglycerides (TG), glucose, insulin, and C-reactive protein (CRP) were measured.

Colorimetric enzymatic-assays (Roche Diagnostics, Monza, Italy) were used to determine serum T-C, LDL-C, HDL-C, and TG levels. The sensitivities of the assays were 3.86 mg/dL (1 mg/dL = 0.03 mmol/L), 3.87 mg/dL (1 mg/dL = 0.03 mmol/L), 3.09 mg/dL (1 mg/dL = 0.03 mmol/L) and 8.85 mg/dL (1 mg/dL = 0.01 mmol/L), respectively.

Serum glucose level was measured by the glucose oxidase enzymatic method (Roche Diagnostics, Monza, Italy). The sensitivity of the method was 2 mg/dL (1 mg/dL = 0.06 mmol/L).

Serum insulin concentration was determined by a chemiluminescent immunometric assay, using a commercial kit (Elecsys Insulin, Roche Diagnostics, Monza, Italy). The sensitivity of the method was 0.2 µIU/mL (1 µU/mL = 7.18 pmol/L).

CRP was measured using an immunoturbidimetric assay (CRP RX, Roche Diagnostics GmbH, Mannheim, Germany). The sensitivity of the method was 0.3 mg/L.

The intra- and inter-assay coefficients of variation (CVs) were the following: 1.1% and 1.6% for T-C, 1.2% and 2.5% for LDL-C, 1.8% and 2.2% for HDL-C, 1.1% and 2.0% for TG, 1.0% and 1.3% for glucose, and 1.5% and 4.9% for insulin.

For each patient, we also calculated the homeostatic model assessment of insulin resistance (HOMA-IR) according to the following formula: (insulin (μIU/mL) × glucose (mmol/L))/22.5 [20].

### 2.5. Blood Pressure

Blood pressure was measured on the right arm, using a sphygmomanometer with appropriate cuff size, with the subject in a seated position and a relaxed condition. The procedure was repeated three times at 10 min intervals between; the means of the three values for SBP and DBP were recorded.

### 2.6. Definition of Metabolic Syndrome

According to the International Diabetes Federation (IDF) criteria for diagnosis of metabolic syndrome in adults [21], obese patients were considered positive for the presence of metabolic syndrome if they had three or more of the following factors: (i) abdominal obesity; (ii) hypertriglyceridemia or specific treatment for this lipid abnormality; (iii) reduced HDL-C levels or specific treatment for this lipid abnormality; (iv) blood (systolic or diastolic) hypertension or treatment of previously diagnosed hypertension; (v) hyperglycemia or previously diagnosed type 2 diabetes mellitus.

### 2.7. Calculation of Framingham Risk Score and Vascular Age

The 2008 Framingham risk score (FRS) assessment was employed to determine the CVD risk and, additionally, the FRS-based vascular age (VA) [22]. 

The FRS algorithm considers age, T-C, HDL-C, SBP, ongoing treatment of hypertension, smoking, and diabetes status and provides sex-specific results. 

VA is defined as the age of a person with the same predicted CV risk, but with all other risk factor levels in the normal ranges. 

### 2.8. Determination of the Epigenetic Age Acceleration

Subjects provided a blood sample at T1 and T2, which was collected in EDTA-containing tubes and immediately stored at −80 °C until assayed for DNAm age. 

Blood samples were thawed, and genomic DNA was extracted using the Wizard Genomic DNA Purification Kit (Promega; Madison, WI, USA) according to the manufacturer’s instructions.

DNAm age was calculated considering the methylation pattern of five CpG sites at five genes (ELOVL2, C1orf132/MIR29B2C, FHL2, KLF14, TRIM59) as reported elsewhere [19]. The DNA samples (500 ng) were plated at a concentration of 25 ng/μL in plates of 96 wells each and were treated with sodium bisulfite using the EZ-96 DNA Methylation-Gold™ Kit (Zymo Research; Irvine, CA, USA) following the manufacturer’s instructions and eluted in 200 μL. Then, 10 μL of bisulfite-treated template DNA was added to 25 μL of GoTaq Hot Start Green Master mix (Promega), 1 μL of the forward primer (10 μM), and 1 μL of the 50 end-biotinylated reverse primer (10 μM) to set up a 50 μL PCR reaction. PCR cycling conditions and primer sequences have been previously reported [19].

Biological (epigenetic) age (Y) was calculated as follows:

Y = 3.26847784751817 + 0.465445549010653 × methC7-ELOVL2 − 0.355450171437202 × methC1-C1orf132 + 0.306488541137007 × methC7-TRIM59 + 0.832684435238792 × methC1-KLF14 + 0.237081243617191 × methC2-FHL2.

### 2.9. Statistical Analysis

The sample size was calculated for epigenetic age acceleration. Based on previous studies of our group on this parameter, we estimated that at least 70 subjects were necessary to demonstrate a mean difference of 15% with type I and II errors set at 0.05 and 0.20. 

Pre- and post-values of demographic, lifestyle, biochemical, clinical characteristics, and CVD outcomes were compared for continuous variables with paired *t*-test or Wilcoxon signed-rank test for paired data as appropriate. Categorical data were compared with the McNemar test for paired data. 

We calculated the correlation between chronological age and DNAm age with Pearson correlation coefficients before and after BWRP.

Epigenetic age acceleration was estimated by regressing DNAm age on chronological age and taking the residuals of the model (the difference between the observed DNAm age and the predicted DNAm age). By using this method, we obtained a measure of epigenetic age acceleration that was independent of chronological age. A positive (>0 yrs) age acceleration means that the subject is biologically older than expected accordingly to chronological age. 

The graphical analysis with boxplots and spaghetti plots was utilized to describe pre- and post-BWRP values of epigenetic age acceleration. We applied Wilcoxon signed-rank test for paired data to investigate the significance of the change of epigenetic age acceleration after BWRP intervention. Furthermore, we replicated this analysis in two subgroups of subjects divided on the epigenetic age acceleration measured at the baseline: ≤0 yrs or >0 yrs. 

Univariate linear regression models were applied to evaluate the associations between the pre-BWRP epigenetic age acceleration and the demographic, lifestyle, biochemical, and clinical characteristics measured at baseline. We applied the same models dividing the population into the two subgroups having a baseline epigenetic age acceleration of ≤0 yrs or >0 yrs. 

A restricted cubic spline function analysis was used to graphically explore the association of CRP levels with epigenetic age acceleration.

Finally, we evaluated the Pearson correlation coefficients between chronological age, DNAm age, and VA before BWRP.

The statistical analyses were performed using SAS software (version 9.4, SAS Institute, Milan, Italy) and *p*-values below 0.05 were considered statistically significant. 

## 3. Results

Seventy-two obese adults (F/M: 43/29; (chronological) age: 51.5 ± 14.5 yrs; BMI: 46.5 ± 6.3 kg/m^2^) were recruited and, after having completed the 3 weeks of BWRP, included in the next statistical analysis. Table 1 summarizes the demographic, lifestyle, biochemical, and clinical characteristics of the entire population at the baseline conditions (at T1, i.e., before the BWRP) and at the end of the intervention (at T2, i.e., after the BWRP). Table S1 (in the Supplementary Material) reports the description of T1 demographic, lifestyle, biochemical, and clinical measures stratified according to epigenetic age acceleration (≤ or >0 yrs).

At the end of the BWRP, when considering the entire population, in addition to weight loss, positive changes in body composition, beneficial metabolic effects, and an improvement in the cardiovascular function and the systemic inflammatory state (Table 1), VA and FRS were significantly reduced (VA (pre- vs. post-BWRP): 41.37 ± 14.81 yrs vs. 38.48 ± 14.80 yrs, *p* < 0.0001; FRS (pre- vs. post-BWRP): 13.88 ± 11.88 vs. 11.76 ± 10.62, *p* < 0.0001).

**Figure 1:**
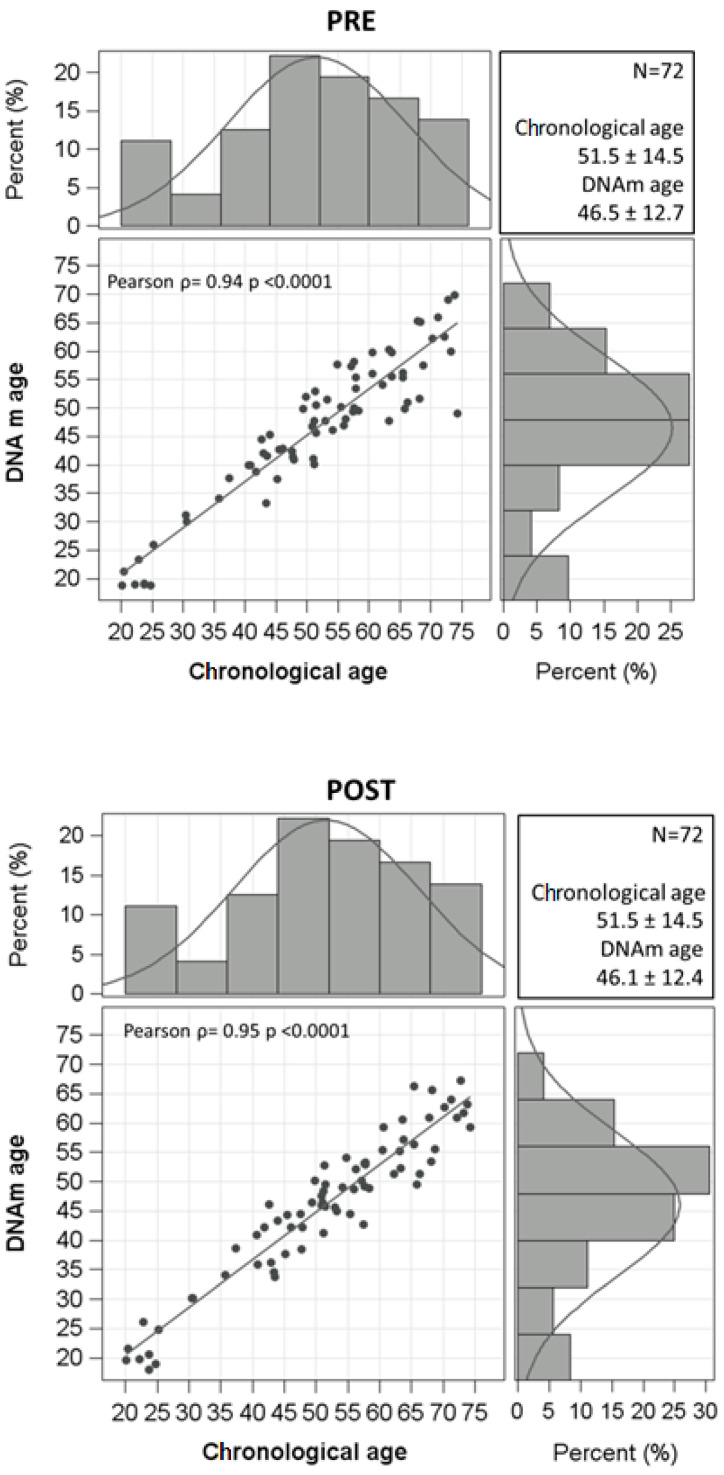
Distribution of chronological age and DNA methylation (DNAm) age. Scatterplots and Pearson’s correlation coefficients for chronological age and DNAm age (estimated by Zbiec–Piekarska formula, pre- and post-BWRP) are reported.

Figure 1 shows population distributions of chronological age and DNAm age at both T1 (pre-BWRP) and T2 (post-BWRP), together with the corresponding Pearson’s correlation coefficients (pre-BWRP: ρ = 0.94 and *p* < 0.0001; post-BWRP: ρ = 0.95 and *p* < 0.0001).

**Figure 2:**
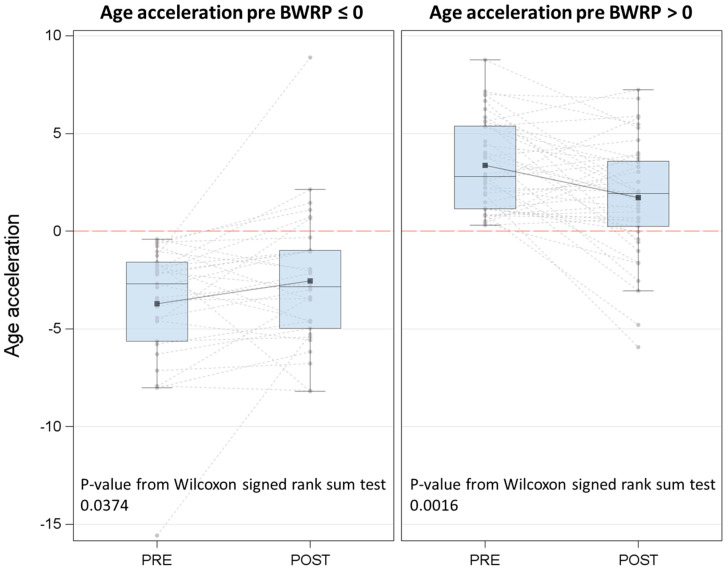
Epigenetic age acceleration: pre- and post-BWRP values are reported as spaghetti and box plots. Population was divided by epigenetic age acceleration at T1 (pre-BWRP: ≤0 or >0 yrs).

When considering the entire population, DNAm age and epigenetic age acceleration did not significantly differ after the BWRP (DNAm age: 46.5 ± 12.7 yrs vs. 46.1 ± 12.4 yrs, *p* = ns; (median) epigenetic age acceleration: 0.79 (−2.12–3.78) yrs vs. 0.36 (−2.92–2.10) yrs, *p* = ns) (Table 1). However, when subdividing the population into two groups based on the epigenetic age acceleration at the baseline (i.e., epigenetic age acceleration ≤ 0 yrs or >0 yrs), the BWRP significantly reduced epigenetic age acceleration in the group having a value > 0 yr (pre- vs. post-BWRP: 2.81 (1.16–5.37) yrs vs. 1.93 (0.23–3.58) yrs, *p* = 0.0016) (improvement), while significantly, but modestly, increasing that in the group having a value ≤ 0 yr (pre- vs. post-BWRP: −2.71 (−5.63–−1.59) yrs vs. −2.86 (−4.97–−0.99) yrs, *p* = 0.0374) (worsening) (Figure 2).

Considering the epigenetic age acceleration at T1 for all participants as a dependent variable, among all the single demographic, lifestyle, biochemical, and clinical characteristics investigated (independent variables), only some markers of systemic inflammation were significantly associated with the epigenetic age acceleration (CRP: β = 1.7746, standard error (SE) = 0.7503 and *p* = 0.0208; NLR: β = 1.6163, SE = 0.7368 and *p* = 0.0376; PLR: β = 0.4326, SE = 0.176 and *p* = 0.0164). 

Applying the same statistical approach in the two groups with epigenetic age acceleration at baseline ≤ 0 yrs or >0 yrs, only in the latter one, the associations of the epigenetic age acceleration with some markers of systemic inflammation deserve to be mentioned, though the statistical significance was not reached (CRP: β = 1.0638, SE = 0.5719 and *p =* 0.0704; PLR: β = 0.2107, SE = 0.1061 and *p* = 0.0540) (Table S2a in the Supplementary Material).

**Figure 3:**
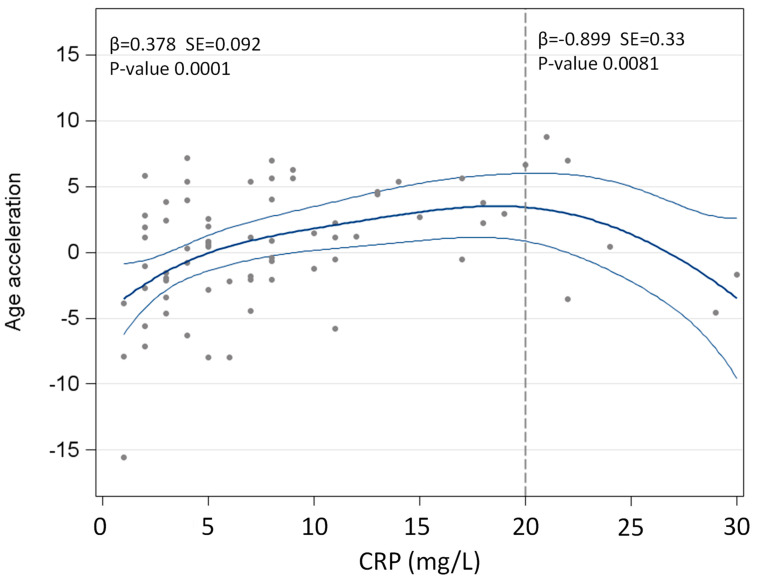
Relationship of epigenetic age acceleration at pre-BWRP with CRP concentrations, evaluated by restricted cubic splines.

After having performed an explorative restricted cubic spline function in order to investigate the relationship between epigenetic age acceleration and CRP levels at T1, a cut-off of CRP equal to 20 mg/L identified two groups of obese subjects having an opposed relationship (Figure 3). In particular, for the individuals with CRP < 20 mg/L the association was positive and statistically significant, so that for every unit increase of CRP there was a 0.378 yr epigenetic age acceleration increment (β = 0.378, SE = 0.092 and *p* = 0.0001), while the opposite was found in subjects with CRP > 20 mg/L (β = −0.899, SE = 0.330 and *p* = 0.0081). This implies that, below or above a specified level, the systemic inflammatory state negatively (with an increase) or positively (with a decrease) influenced the epigenetic age acceleration (Figure 3). 

In order to evidence a different impact of the BWRP (pre- vs. post-BWRP), all the demographic, lifestyle, biochemical, and clinical parameters were compared within the single group with the epigenetic age acceleration ≤ 0 yrs or > 0 yrs. In both groups, the BWRP significantly reduced (pre- vs. post-BWRP) SBP (group with epigenetic age acceleration ≤0 yrs: 137.4 ± 2.0 mmHg vs. 131.1 ± 2.0 mmHg, *p* = 0.0315; group with epigenetic age acceleration >0 yrs: 135.5 ± 2.0 mmHg vs. 128.2 ± 2.0 mmHg, *p* = 0.0146), DBP (group with epigenetic age acceleration ≤0 yrs: 85.2 ± 1.7 mmHg vs. 79.4 ± 1.7 mmHg, *p* = 0.0197; group with epigenetic age acceleration >0 yrs: 84.8 ± 1.3 mmHg vs. 79.2 ± 1.3 mmHg, *p* = 0.0037), glucose (group with epigenetic age acceleration ≤0 yrs: 5.6 ± 1.0 mmol/L vs. 5.2 ± 1.0 mmol/L, *p* = 0.0076; group with epigenetic age acceleration >0 yrs: 6.1 ± 1.0 mmol/L vs. 5.4 ± 1.3 mmol/L, *p* = 0.0089), T-C (group with epigenetic age acceleration ≤0 yrs: 195.3 ± 6.3 mg/dL vs. 172.4 ± 6.3 mg/dL, *p* = 0.0156; group with epigenetic age acceleration >0 yrs: 192.4 ± 5.9 mg/dL vs. 169.0 ± 5.9 mg/dL, *p* = 0.0077), LDL-C (group with epigenetic age acceleration ≤0 yrs: 126.9 ± 5.8 mg/dL vs. 108.2 ± 5.8 mg/dL, *p* = 0.0297; group with epigenetic age acceleration >0 yrs: 126.1 ± 5.4 mg/dL vs. 106.6 ± 5.4 mg/dL, *p* = 0.0144) and NLR (group with epigenetic age acceleration ≤ 0 yrs: 1.9 ± 0.1 vs. 1.3 ± 0.1, *p* < 0.0001; group with epigenetic age acceleration >0 yrs: 2.3 ± 0.1 vs. 1.7 ± 0.1, *p* = 0.0010). Only in the group with epigenetic age acceleration >0 yrs, the BWRP significantly reduced (pre- vs. post-BWRP) HOMA-IR (7.2 ± 0.6 vs. 5.4 ± 0.6, *p* = 0.0317), HDL-C (48.5 ± 1.6 mg/dL vs. 43.4 ± 1.6 mg/dL, *p* = 0.0299) and CRP (7 ± 11 mg/L vs. 5 ± 11 mg/L, *p* = 0.0329) (see Table S2b in the Supplementary Material).

Chronological age was significantly correlated with VA (ρ = 0.78 and *p* < 0.0001). Importantly, there was a significant correlation between DNAm age and VA (ρ = 0.74 and *p* < 0.0001), indicating the existence of a relationship between a surrogate marker of biological aging and a validated index of CVD risk. 

## 4. Discussion

BMI has been reported to be associated with epigenetic age acceleration, particularly in severely obese subjects [23]. Diet, exercise, education, and lifestyle are factors influencing epigenetic age acceleration [24].

In the present study, carried out in a cohort of severely obese adults who underwent a 3-week multidisciplinary BWRP, including diet and exercise, the epigenetic age acceleration was shown to be decreased at the end of the weight-losing intervention only in the group with an epigenetic age acceleration >0 yrs, yet remaining unchanged in the entire population or modestly increased in the group with an epigenetic age acceleration ≤0 yrs.

Unfortunately, conflicting results have been published regarding the effect of weight loss on epigenetic age acceleration. A study carried out in severely obese patients treated with bariatric surgery showed a clear-cut improvement of several cardiometabolic parameters within a 9-month post-surgical period; however, the marked weight loss did not reverse epigenetic age acceleration, which was measured in the liver [25]. In a previous study, exercise was reported to induce genome-wide changes in DNAm of adipose tissue, without changing BMI at the end of the intervention [26]. However, after a next re-analysis of the dataset of this work through a different algorithm, epigenetic age acceleration was demonstrated not to be affected by the exercise-based intervention [25]. In contrast with this view of a presumptively irreversible obesity-related epigenetic aging, CR was shown to slow the epigenetic clock in an animal model (i.e., mice liver) [27]. Moreover, in the CENTRAL MRI trial, an 18-month lifestyle weight-losing intervention was demonstrated to attenuate DNAm aging, particularly in the group with a >5% weight loss [28]. Finally, an 8-week treatment consisting of diet and some changes in lifestyle was reported to be associated with a 3.23 yrs decrease in salivary DNAm age when administered in male adults compared with untreated subjects [29].

To date, we are unable to explain the reasons for the discrepancy among these studies, including ours. We can only tentatively suppose that the BWRP-induced rejuvenation that was uniquely observed in the group with an epigenetic age acceleration > 0 yrs is related to the “specific” characteristics of our BWRP, which, however, did reduce BMI in both groups (with epigenetic age acceleration ≤ or >0 yrs). Furthermore, in the present study, (baseline values of and changes in) epigenetic age acceleration and BMI were not associated, implying that weight loss does not play a pivotal role in the BWRP-induced anti-aging effect and that we should search for other molecular and cellular mechanisms.

The subdivision of our obese population into the two groups based on the epigenetic age acceleration might have disclosed, differently from other previously cited studies, the existence of obese subjects having “biological” characteristics that make them more responsive to the BWRP-induced anti-aging effect. Thus, identification of such biological characteristics might be of fundamental importance to understanding our results.

In the present study, epigenetic age acceleration was associated with CRP (in the entire population and, though not significantly, in the group with an epigenetic age acceleration > 0 yrs) as well as other markers of systemic inflammation. Furthermore, levels of CRP below 20 mg/L identified a group of obese subjects for whom epigenetic age acceleration was directly associated with CRP levels. Though CRP is a surrogate of systemic inflammation, the biological substrate that links aging with obesity might be the systemic inflammation itself [30]. In the present study, 3 weeks of BWRP was capable of reducing CRP levels, particularly in the group with an epigenetic age acceleration > 0 yrs.

Therefore, based on the previous considerations, we suppose the following sequence of pathophysiological events: (1) in some (environmentally and/or genetically predisposed?) obese subjects, chronic systemic inflammation promotes a DNA destabilization, owing to the detrimental pro-aging effects of the enhanced release of reactive oxygen species (ROS), the mitochondrial dysfunction, and the aberrant activation of proliferative signaling [7]; (2) a counter-regulatory epigenetic DNA remodeling occurs, including changes in DNA methylation/demethylation, a biological phenomenon that can be evaluated, though crudely, by means of DNAm age [6]; and (3) the BWRP-induced anti-inflammatory action might reverse, at least partially, these molecular and cellular mechanisms, explaining its presumptive effect of rejuvenation (i.e., post-BWRP decrease in epigenetic age acceleration) [18].

Some epidemiological studies have shown a strong association of DNAm age with time to death (due to all-cause mortality), cause-specific death, coronary heart disease, and disease-free status, suggesting the existence of a link between epigenetic clock and biological aging, giving rise to a unified theory of life course [31,32]. In this regard, the present study demonstrates a correlation of DNAm age with FRS and VA, which represent two internationally validated methods for CVD risk assessment [22]. Importantly, our 3-week BWRP reduced both FRS and VA [33,34], a finding that was congruent with the post-BWRP decrease in epigenetic age acceleration. Though these statistical associations do not imply a relationship of cause–effect, we argue that any intervention aimed at reducing epigenetic age acceleration such as BWRP should result in a parallel improvement of CVD risk (or vice versa) and prolongation of lifespan and healthy status [35,36]. In the specific context of the present study, obese subjects with an epigenetic age acceleration > 0 yrs should maximally benefit from the BWRP in terms of both CVD risk and life expectancy. 

Before closing, some limitations should be mentioned.

First of all, we have characterized a group of obese adults having an epigenetic age acceleration >0 yrs that might benefit from the BWRP-induced anti-aging effect. Since we have found no relevant differences in demographic, lifestyle, biochemical, and clinical parameters between the two groups at different epigenetic age acceleration (i.e., > or ≤0 yrs), this implies the need to identify the environmental and/or genetic factors underlying the different epigenetic age acceleration that characterizes the single obese subject. Further studies are mandatory to solve this issue. This information might be also useful to better tailor interventions for weight loss in obese subjects, particularly those with an epigenetic age acceleration ≤ 0 yrs.

Second, in the present study, we have demonstrated that, above a cut-off of 20 mg/L, CRP levels become inversely associated with epigenetic age acceleration. This might be a “paradoxical” statistical artifact (due to the six out of 72 subjects falling in this group), but, in our opinion, because of the association of epigenetic loci in its gene with inflammation and CVD [37], CRP might become the “perpetrator and victim” of the same obesity-related DNA destabilization previously described [38,39,40,41,42].

Finally, DNAm age, measured with different methods, is only a surrogate of aging [6]. Therefore, the results deriving from DNAm-age-based studies should be cautiously interpreted.

## 5. Conclusions

A 3-week multidisciplinary BWRP is capable of reducing the epigenetic age acceleration in obese adults, being the BWRP-induced rejuvenation particularly evident in subjects with an epigenetic age acceleration > 0 yrs. Based on the BWRP-induced decrease in CRP levels, chronic systemic inflammation seems to play a role in mediating obesity-related epigenetic remodeling and biological aging. Thus, due to the strong association of CVD risk with the epigenetic clock and morbidity/mortality, any effort should be made to reduce the low-grade chronic inflammatory state in obesity (e.g., through the administration of a long-term BWRP) [43].

## Figures and Tables

**Table 1:** Demographic, lifestyle, biochemical, and clinical characteristics of participants (N = 72) at the study: comparison pre- vs. post-BWRP.

Characteristics	Pre	Post	*p*-Value
Age, yrs	51.5 ± 14.5		-
DNAm age, yrs	46.5 ± 12.7	46.1 ± 12.4	0.2809
Age acceleration, years	0.79 (−2.12–3.78)	0.36 (−2.92–2.10)	0.3078
Gender			
*Males*	29 (40.28%)		-
*Females*	43 (59.7%)		
Smoking status			
*Never smoker*	37 (51.4%)		-
*Former smoker*	18 (25.0%)		
*Current smoker*	17 (23.6%)		
Alcohol consumption			
*Yes*	8 (11.1%)		-
*No*	58 (80.6%)		
*Occasionally*	6 (8.3%)		
Education			
*Primary school or less*	7 (9.7%)		-
*Secondary school*	28 (38.9%)		
*High school*	29 (40.3%)		
*University*	8 (11.1%)		
Occupation			
*Employed*	36 (50.0%)		-
*Unemployed*	14 (19.4%)		
*Retired*	14 (19.4%)		
*Housewife*	5 (6.9%)		
*Student*	3 (4.2%)		
Physical activity levels			
*Sedentary*	58 (80.6%)		-
*Active*	13 (18.1%)		
*Sporty*	1 (1.4%)		
BMI, kg/m^2^	46.5 ± 6.3	45 ± 5.9	<0.0001
Weight, kg	117.2 (107.7–137.9)	114 (103.8–133.5)	<0.0001
Height, m	1.64 ± 0.1		-
WC, cm	125.9 ± 13.4	121.2 ± 12.1	<0.0001
FFM kg	53.2 (47.9–67.5)	52.3 (48.7–66.3)	0.0137
FFM %	46.5 ± 5.8	47.8 ± 7.0	0.0165
FM kg	67.1 ± 15.8	63.6 ± 15.4	<0.0001
FM %	53.5 ± 5.8	52.9 ± 6.4	0.3675
Heart rate, bpm	80.0 ± 11.7	76.5 ± 9.2	0.0171
Systolic blood pressure, mmHg	136.3 ± 13.0	129.4 ± 11.3	0.0001
Diastolic blood pressure, mmHg	84.9 ± 9.7	79.2 ± 7.4	0.0001
Anti-hypertensive drugs			
*Yes*	37 (51.4%)	35 (48.6%)	1
*No*	35 (48.6%)	37 (51.4%)	
Glucose, mmol/L	5.6 (5.2–6.0)	5.3 (4.9–5.5)	<0.0001
Insulin, mIU/L	20.5 (13.5–30.6)	19.4 (14.3–24.6)	<0.0001
HOMA-IR	6.6 ± 4.2	5.0 ± 2.5	0.0023
HbA1c, %	5.7 (5.5–5.9)	5.6 (5.4–5.8)	<0.0001
Diabetes			
*Yes*	11 (15.3%)	10 (13.9%)	<0.0001
*No*	61 (84.7%)	62 (86.1%)	
Anti-diabetic medications			
*Yes*	12 (16.7%)	13 (18.1%)	<0.0001
*No*	60 (83.3%)	59 (81.9%)	
Metabolic syndrome			
*Yes*	44 (61.1%)	39 (54.2%)	0.2100
*No*	28 (38.9%)	33 (45.8%)	
Total cholesterol, mg/dL	193.7 ± 36.1	170.4 ± 37.0	<0.0001
HDL-C, mg/dL	49.4 ± 11.4	44.7 ± 10.6	<0.0001
LDL-C, mg/dL	126.4 ± 34.0	107.3 ± 32.5	<0.0001
Triglyceride, mg/dL	145.5 ± 53.7	131.9 ± 44.3	0.0063
Lipid-lowering drugs			
*Yes*	9 (12.5%)	12 (16.7%)	<0.0001
*No*	63 (87.5%)	60 (83.3%)	
CRP *, mg/L	7 (4–11)	4 (2–8.5)	<0.0001
Neutrophils, %	59 ± 7.5	51.1 ± 7.7	<0.0001
Lymphocytes, %	30.1 ± 6.9	36.1 ± 7.8	<0.0001
Monocytes, %	7.8 ± 1.8	9.4 ± 2.5	<0.0001
Eosinophils, %	2.2 (1.5–3.2)	2.4 (1.5–3.5)	0.0027
Basophils, %	0.55 (0.4–0.7)	0.6 (0.4–0.8)	0.0090
Platelets, ×10^5^/mm^3^	259.1 ± 55.5	238.3 ± 54.8	<0.0001
NLR	2.11 ± 0.70	1.53 ± 0.58	<0.0001
PLR	125.4 ± 41.4	115.3 ± 44.0	0.0150
AST, U/L	21 (18–28)		-
ALT, U/L	22 (16.5–36)	28.5 (20–50.5)	<0.0001
Gamma GT, U/L	25.5 (19.5–36)	23 (16–30.5)	<0.0001
Creatinine, mg/dL	0.80 ± 0.16		-
VA, yrs	41.37 ± 14.81	38.48 ± 14.80	<0.0001
FSR, %	13.88 ± 11.88	11.76 ± 10.62	<0.0001

For normal distribution, values are expressed as mean ± standard deviation, and, when possible, we applied paired *t*-test. When not normally distributed, values are expressed as median (Q1, Q3), and we applied Wilcoxon signed-rank test for paired data. Categorical data are reported as frequencies and percentage; for these, we applied McNemar test for paired data. For abbreviations see the text. * CRP range (min–max) in all datasets: 1–30 mg/L.

## Supplementary Materials

The following supporting information can be downloaded at: https://www.mdpi.com/article/10.3390/jcm11164677/s1, Table S1: Demographic, lifestyle, biochemical and clinical characteristics of participants (N = 72) at T1 (pre-BWRP): comparisons between the two groups of epigenetic age acceleration; Table S2. (a): Associations between demographic, lifestyle, biochemical and clinical characteristics and epigenetic age acceleration (at T1, pre-BWRP), divided by epigenetic age acceleration (≤0 vs. >0 yrs); (b): Comparisons between pre- vs. post-BWRP demographic, lifestyle, biochemical and clinical characteristics within each group, divided by epigenetic age acceleration (≤0 vs. >0 yrs).

## Abbreviations

ALT, alanine transaminase; ASP, aspartate transaminase; BMI, body mass index; bpm, beats per minute; BWRP, body weight reduction program; CI, confidence interval; CRP, C reactive protein; CV, coefficient of variation; DBP, diastolic blood pressure; DNAm, DNA methylation; F, female; FRS, Framingham risk score; gamma-GT, gamma-glutamyl transferase; HC, hip circumference; HDL-C, high-density lipoprotein cholesterol; HOMA-IR, homeostatic model assessment for insulin resistance; HR, heart rate; IGF-1, insulin-like growth factor 1; IQR, interquartile range; LDL-C, low-density lipoprotein cholesterol; M, male; mTOR, mammalian target of rapamycin; NLR, neutrophils to lymphocytes ratio; PLR, platelets to lymphocytes ratio; ROS, reactive oxygen species; SBP, systolic blood pressure; SD, standard deviation; SE, standard error; T-C, total cholesterol; TG, triglycerides; VA, vascular age; WC, waist circumference; yr, year.
