# Supplementary material for: Effect of a 3-Week Multidisciplinary Body Weight Reduction Program on the Epigenetic Age Acceleration in Obese Adults
Source: J Clin Med. 2022 Aug 10;11(16):4677. doi: 10.3390/jcm11164677 (PMC9410133; doi:10.3390/jcm11164677)
Supplement: Supplementary file 1 [file jcm-11-04677-s001.zip › jcm-1808447-supplementary.pdf]

TABLE S1 - Demographic, lifestyle, biochemical and clinical characteristics of participants (N=72) at T1 (pre-BWRP): comparisons between the two groups of epigenetic age acceleration.

	**AGE ACCELERATION ≤ 0 N=31**	**AGE ACCELERATION > 0 N=41**	**P-value**
Age, years	53.15±15.86	50.32±13.5	0.4166
DNAm age	43.8±12.83	48.6±12.27	0.3629
Weight, kg	113.6 [105;132.5]	119.1 [109;137.9]	0.4448
Height, m	1.63±0.11	1.64±0.1	0.4857
BMI, *kg/m^2*	46.1±6.56	46.85±6.13	0.6185
WC., cm	124.97±13.81	126.56±13.11	0.6194
FFM kg	51.56 [46.35;66.7]	54.32 [49.82;67.8]	0.3421
FFM %	46.24±4.92	46.78±6.47	0.7026
FM kg	65.78±15.31	68.1±16.32	0.5437
FM %	53.76±4.92	53.23±6.47	0.7026
Systolic blood pressure, mmHg	137.42±11.32	135.49±14.13	0.5345
Diastolic blood pressure, mmHg	85.16±11.58	84.76±8.14	0.8621
Heart rate, bpm	79.35±10.65	80.44±12.46	0.6987
Glucose, mmol/L	5.44 [5.16;5.83]	5.77 [5.22;6.16]	0.1378
Insulin, mU/L	17.2 [13.6;26.3]	20.7 [13.4;35.7]	0.2353
HOMA-IR	5.66±3.4	7.23±4.68	0.1187
Total cholesterol, mg/dL	195.29±33.54	192.44±38.21	0.7423
HDL-C, mg/dL	50.55±12.25	48.51±10.81	0.4575
LDL-C, mg/dL	126.9±30.16	126.05±36.95	0.9167
Triglyceride, mg/dL	152.48±65.29	140.22±43.03	0.3407
HbA1c, %	5.7 [5.4;5.8]	5.8 [5.6;6.1]	0.2353
CRP, mg/L	5 [3;8]	8 [05;13]	**0.0408**
Neutrophils, %	57.35±6.8	60.27±7.74	0.1003
Lymphocytes, *%*	31.62±6.6	28.89±6.88	0.0946
Monocytes, *%*	8.06±1.9	7.62±1.62	0.2945
Eosinophils, *%*	2.4±1.17	2.63±2.07	0.5741
Basophils, *%*	0.56±0.2	0.58±0.19	0.7285
Platelets, $\times10^5/mm^3$	252.74±50.4	263.02±59.03	0.4389
PLR	8.23±1.98	9.58±3.37	**0.0369**
NLR	1.91±0.51	2.26±0.79	**0.0264**
AST,U/L	25.16±10.41	24.61±14.39	0.5176
ALT,U/L	21 [15;37]	22 [17;35]	0.8943
Gamma GT, U/L	25 [18;36]	26 [20;36]	0.7253
Creatinine, mg/dL	0.78±0.16	0.82±0.15	0.2342

For normal distribution, values are expressed as mean ± standard deviation, and we applied t-test. When not normal distributed, values are expressed as median [Q1; Q3], and we applied Wilcoxon signed rank test.

TABLE S2a – Associations between demographic, lifestyle, biochemical and clinical characteristics and epigenetic age acceleration (at T1, pre-BWRP), divided by epigenetic age acceleration (≤0 vs. >0 yrs).

**Dependent variable**	**Acceleration Age ≤ 0 N=31**			**Acceleration Age > 0 N=41**		
	**β**	**SE**	**P-value**	**β**	**SE**	**P-value**
BMI, *kg/m²*	0.1155	0.0889	0.2040	0.0032	0.0612	0.9579
Weight, kg	0.0197	0.0228	0.3949	-0.0103	0.0160	0.5223
Height, m	-0.9571	5.3966	0.8605	-3.8640	3.8222	0.3183
WC, cm	0.0199	0.0432	0.6482	0.0300	0.0282	0.2932
FFM kg	-0.0063	0.0433	0.8860	-0.0463	0.0296	0.1269
FFM %	-0.2233	0.1146	0.0612	-0.0707	0.0576	0.2273
FM kg	0.0618	0.0374	0.1098	0.0030	0.0233	0.8986
FM %	0.2233	0.1146	0.0612	0.0707	0.0576	0.2273
Heart rate, bpm	0.0263	0.0561	0.6429	-0.0359	0.0295	0.2312
Systolic blood pressure, mmHg	0.0421	0.0524	0.4275	-0.0218	0.0263	0.4122
Diastolic blood pressure, mmHg	0.0439	0.0511	0.3974	-0.0145	0.0460	0.7552
Glucose, mmol/L	0.2701	0.6702	0.6899	-0.0596	0.1668	0.7226
Insulin, mU/L	-0.0234	0.0481	0.6305	-0.0136	0.0264	0.6080
HOMA-IR	-0.0355	0.1759	0.8413	-0.0329	0.0799	0.6832
HbA1c, %	0.547	0.4204	0.1974	-0.0159	0.2369	0.9467
Total cholesterol, mg/dL	-0.0070	0.0178	0.6978	0.0085	0.0097	0.3883
HDL, mg/dL	-0.0228	0.0487	0.6437	0.0521	0.0337	0.1298
LDL, mg/dL	-0.0056	0.0198	0.7815	0.0050	0.0101	0.6240
Triglycerides, mg/dL	-0.0031	0.0092	0.7392	-0.0009	0.0087	0.9209
**PCR, mg/dL**	1.0389	0.7745	0.1902	**1.0638**	**0.5719**	**0.0704**
Neutrophils, %	-0.0336	0.0879	0.7048	0.0525	0.0477	0.2776
Lymphocytes, %	0.0456	0.0904	0.6181	-0.0660	0.0535	0.2249
Monocytes, %	-0.1062	0.3148	0.7383	0.0067	0.2310	0.9770
Eosinophils, %	-0.0764	0.5135	0.8828	-0.0172	0.1810	0.9250
Basophils, %	1.5237	3.0468	0.6208	0.7215	1.9702	0.7162
Platelets, $\times 10^5/mm^3$	-0.0008	0.0119	0.9479	0.0053	0.0063	0.4009
**NLR**	-0.3747	1.1841	0.7539	0.6247	0.4618	0.1839
**PLR**	-0.03436	0.3030	0.9105	**0.2107**	**0.1061**	**0.0540**
AST (U/L)	0.0067	0.0576	0.9081	-0.0024	0.0261	0.9272
ALT (U/L)	0.0058	0.0195	0.7675	-0.0113	0.0143	0.4335
gamma GT	0.0085	0.0151	0.5798	-0.0045	0.0074	0.5453
**Creatinine, mg/dL**	**-10.3107**	**3.2680**	**0.0037**	2.2239	2.4305	0.3658

TABLE S2b – Comparisons between pre- vs. post-BWRP demographic, lifestyle, biochemical and clinical characteristics within each group, divided by epigenetic age acceleration (≤0 vs. >0 yrs).

		Acceleration Age ≤0 N=31					Acceleration Age > 0 N=41				
**outcome**	**time**	**Estimate**	**se**	**95% CI**		**P-value**	**Estimate**	**se**	**95% CI**		**P-value**
Weight, kg *	**pre**	120.44	1.03	112.61	128.81		125.22	1.03	118.68	132.11	
	**post**	116.37	1.03	108.81	124.47	0.4667	121.25	1.03	114.92	127.93	0.3961
	**diff**	-4.06	1.04				-3.96	1.04			
BMI, kg/m^2	**pre**	46.10	1.15	43.76	48.44		46.85	0.93	44.98	48.72	
	**post**	44.53	1.15	42.19	46.87	0.3410	45.35	0.93	43.48	47.22	0.2578
	**diff**	-1.57	1.62				-1.50	1.31			
WC, cm	**pre**	124.97	2.32	120.22	129.71		126.56	1.98	122.56	130.56	
	**post**	120.13	2.32	115.39	124.87	0.1511	122.10	2.00	118.05	126.15	0.1211
	**diff**	-4.84	3.28				-4.46	2.81			
FFM kg *	**pre**	55.39	1.04	50.78	60.41		58.12	1.03	54.66	61.80	
	**post**	55.96	1.04	51.24	61.12	0.8655	56.83	1.03	53.45	60.43	0.6032
	**diff**	0.58	1.06				-1.29	1.04			
FFM %	**pre**	46.24	1.15	43.89	48.59		46.78	1.03	44.69	48.86	
	**post**	48.64	1.17	46.25	51.03	0.1531	47.22	1.03	45.13	49.30	0.7631
	**diff**	2.40	1.64				0.44	1.46			
FM kg	**pre**	65.78	2.73	60.20	71.36		68.10	2.52	63.00	73.21	
	**post**	61.25	2.77	55.58	66.93	0.2539	65.39	2.52	60.29	70.50	0.4526
	**diff**	-4.53	3.89				-2.71	3.57			
FM %	**pre**	53.76	1.01	51.69	55.84		53.23	1.03	51.14	55.31	
	**post**	53.13	1.03	51.02	55.24	0.6675	52.78	1.03	50.70	54.87	0.7631
	**diff**	-0.63	1.45				-0.44	1.46			
SBP, mmHg	**pre**	137.42	1.97	133.39	141.45	0.0315	135.49	2.03	131.39	139.58	0.0146

	**post**	131.13	1.97	127.10	135.16		128.17	2.03	124.07	132.27	
	**diff**	-6.29	2.79				-7.32	2.87			
DBP, mmHg	**pre**	85.16	1.67	81.76	88.56	0.0197	84.76	1.29	82.15	87.36	0.0037
	**post**	79.35	1.67	75.95	82.76		79.15	1.29	76.54	81.75	
	**diff**	-5.81	2.36				-5.61	1.82			
HR, bpm	**pre**	79.35	1.87	75.54	83.17	0.1496	80.44	1.66	77.08	83.80	0.1917
	**post**	75.45	1.87	71.64	79.26		77.32	1.66	73.96	80.68	
	**diff**	-3.90	2.64				-3.12	2.35			
Glucose, mg/dL *	**pre**	101.37	1.02	97.18	105.74	0.0076	109.27	1.03	102.44	116.56	0.0089
	**post**	93.24	1.02	89.39	97.26		96.50	1.03	90.47	102.93	
	**diff**	-8.13	1.05				-12.77	1.05			
Glucose, mmol/L*	**pre**	5.63	1.02	5.39	5.87	0.0076	6.06	1.03	5.69	6.47	0.0089
	**post**	5.17	1.02	4.96	5.40		5.36	1.03	5.02	5.71	
	**diff**	-0.45	0.33				-0.71	1.05			
Insulin, mU/L *	**pre**	18.89	1.10	15.51	22.99	0.5616	22.20	1.08	18.97	25.99	0.4071
	**post**	17.43	1.10	14.32	21.23		20.25	1.08	17.30	23.70	
	**diff**	-1.45	1.11				-1.96	1.12			
HOMA-IR	**pre**	5.66	0.51	4.61	6.71	0.1325	7.23	0.59	6.03	8.42	0.0317
	**post**	4.53	0.51	3.48	5.58		5.36	0.59	4.17	6.56	
	**diff**	-1.13	0.73				-1.86	0.84			
HbA1c, % *	**pre**	5.73	1.07	5.03	6.53	0.5168	6.02	1.03	5.71	6.36	0.2659
	**post**	6.08	1.07	5.33	6.93		5.77	1.03	5.46	6.10	
	**diff**	0.35	1.09				-0.25	1.04			
Total cholesterol, mg/dL	**pre**	195.29	6.32	182.39	208.19	0.0156	192.44	5.91	180.49	204.39	0.0077
	**post**	172.39	6.32	159.48	185.29		168.95	5.91	157.00	180.90	
	**diff**	-22.90	8.93				-23.49	8.36			
HDL, mg/dL	**pre**	50.55	2.15	46.16	54.94	0.1815	48.51	1.60	45.28	51.75	0.0299
	**post**	46.39	2.15	41.99	50.78		43.41	1.60	40.18	46.65	
	**diff**	-4.16	3.04				-5.10	2.26			
LDL, mg/dL	**pre**	126.90	5.78	115.09	138.72	0.0297	126.05	5.37	115.19	136.91	0.0144

	**post**	108.23	5.78	96.41	120.04		106.61	5.37	95.75	117.47	
	**diff**	-18.68	8.18				-19.44	7.60			
Triglycerides, mg/dL	**pre**	152.48	10.47	131.11	173.86	0.2671	140.22	6.45	127.18	153.26	0.2270
	**post**	135.74	10.47	114.36	157.12		129.02	6.45	115.98	142.06	
	**diff**	-16.74	14.80				-11.20	9.12			
Neutrophils, %	**pre**	57.35	1.23	54.84	59.87	0.0000	60.27	1.20	57.83	62.71	0.0002
	**post**	48.47	1.23	45.96	50.99		53.17	1.22	50.70	55.64	
	**diff**	-8.88	1.74				-7.10	1.71			
Lymphocytes, %	**pre**	31.62	1.22	29.12	34.12	0.0005	28.89	1.17	26.53	31.26	0.0022
	**post**	38.35	1.22	35.85	40.85		34.36	1.18	31.97	36.76	
	**diff**	6.73	1.73				5.47	1.67			
Monocytes, %	**pre**	8.06	0.43	7.19	8.93	0.0051	7.62	0.30	7.01	8.24	0.0040
	**post**	9.88	0.43	9.01	10.75		8.95	0.31	8.32	9.57	
	**diff**	1.82	0.60				1.33	0.43			
Eosinophils, %*	**pre**	2.09	1.11	1.70	2.57	0.4328	2.15	1.10	1.77	2.61	0.4429
	**post**	2.35	1.11	1.91	2.89		2.39	1.10	1.96	2.91	
	**diff**	0.25	1.14				0.24	1.15			
Basophils, %	**pre**	0.56	0.04	0.49	0.64	0.2920	0.58	0.15	0.28	0.88	0.2512
	**post**	0.62	0.04	0.54	0.70		0.83	0.15	0.52	1.13	
	**diff**	0.06	0.05				0.25	0.21			
CRP, mg/dL *	**pre**	0.50	1.18	0.35	0.70	0.0612	0.74	1.13	0.58	0.96	0.0329
	**post**	0.31	1.18	0.22	0.44		0.50	1.13	0.39	0.65	
	**diff**	-0.18	1.15				-0.24	1.19			
NLR	**pre**	1.91	0.08	1.74	2.08	<0.0001	2.26	0.11	2.03	2.49	0.0010
	**post**	1.33	0.08	1.16	1.50		1.69	0.11	1.46	1.92	
	**diff**	-0.58					-0.57				
ALT (U/L) *	**pre**	25.98	1.13	20.36	33.14	0.2822	25.45	1.11	20.67	31.34	0.1090
	**post**	31.25	1.13	24.49	39.87		32.31	1.11	26.24	39.79	
	**diff**	5.27	1.14				6.86	1.16			
gamma GT *	**pre**	26.98	1.12	21.59	33.72	0.1927	28.55	1.10	23.41	34.82	0.2137

**post**	21.96	1.12	17.57	27.45	23.95	1.10	19.64	29.21
**diff**	-5.02	1.16			-4.60	1.15		

* GOEMETRIC MEAN
